# Predictive biomarkers for sacituzumab govitecan efficacy in Trop-2-expressing triple-negative breast cancer

**DOI:** 10.18632/oncotarget.27766

**Published:** 2020-10-27

**Authors:** Thomas M. Cardillo, Diane L. Rossi, Maria B. Zalath, Donglin Liu, Roberto Arrojo, Robert M. Sharkey, Chien-Hsing Chang, David M. Goldenberg

**Affiliations:** ^1^Immunomedics, Inc., Morris Plains, NJ 07950, USA; ^2^Currently employed with FrontAim Biomedicines Inc., Princeton, NJ 08540, USA; ^3^Current address: Center for Molecular Medicine and Immunology, Mendham, NJ 07945, USA; ^*^At the time the work was conducted, these authors were employees of Immunomedics, Inc., Morris Plains, NJ 07950, USA; ^#^At the time the work was conducted, this author was Chairman and Chief Scientific Officer of Immunomedics, Inc., Morris Plains, NJ 07950, USA

**Keywords:** sacituzumab govitecan, Trop-2, biomarker, RAD51, triple-negative breast cancer

## Abstract

Sacituzumab govitecan (SG) is an antibody-drug conjugate composed of a humanized anti-Trop-2 IgG antibody conjugated *via* a hydrolysable linker to SN-38, the topoisomerase I-inhibitory active component of irinotecan. We investigated whether Trop-2-expression and homologous recombination repair (HRR) of SN-38-mediated double-strand DNA (dsDNA) breaks play a role in the sensitivity of triple-negative breast cancer (TNBC) to SG. Activation of HRR pathways, as evidenced by Rad51 expression, was assessed in SG-sensitive cell lines with low and moderate Trop-2-expression (SK-MES-1 squamous cell lung carcinoma and HCC1806 TNBC, respectively), compared to a low Trop-2-expressing, less SG-sensitive TNBC cell line (MDA-MB-231). Further, two Trop-2-transfectants of MDA-MB-231, C13 and C39 (4- and 25-fold higher Trop-2, respectively), were treated in mice with SG to determine whether increasing Trop-2 expression improves SG efficacy. SG mediated >2-fold increase in Rad51 in MDA-MB-231 but had no effect in SK-MES-1 or HCC1806, resulting in lower levels of dsDNA breaks in MDA-MB-231. SG and saline produced similar effects in parental MDA-MB-231 tumor-bearing mice (median survival time (MST) = 21d and 19.5d, respectively). However, in mice bearing higher Trop-2-expressing C13 and C39 tumors after Trop-2 transfection, SG provided a significant survival benefit, even compared to irinotecan (MST = 97d *vs.* 35d for C13, and 81d *vs.* 28d for C39, respectively; *P* < 0.0007). These results suggest that SG could provide better clinical benefit than irinotecan in patients with HRR-proficient tumors expressing high levels of Trop-2, as well as to patients with HRR-deficient tumors expressing low/moderate levels of Trop-2.

## INTRODUCTION

In recent years, there has been an increased focus on personalized cancer therapy [1]. One important aspect is the identification of key biomarkers that support a given treatment plan [1–5]. In breast cancer, early research first identified estrogen and progesterone receptor expression as a guide for hormone therapy. Likewise, human epidermal growth factor receptor-2 (HER2) expression has been used since 1998 as a positive biomarker for anti-HER2 therapies [4]. In triple-negative breast cancer (TNBC), targeted therapies based on overexpressed growth factor receptors (e.g., anti-epidermal growth factor receptor (EGFR) antibodies), loss of tumor suppressors (e.g., AKT inhibitors), and genomic instability (e.g., poly ADP ribose polymerase (PARP) inhibitors) have been utilized as strategies to improve therapeutic outcomes [5].

Sacituzumab govitecan (SG; Trodelvy™) is an antibody-drug conjugate (ADC) that specifically targets human trophoblast cell-surface antigen-2 (Trop-2) *via* the humanized anti-Trop-2 antibody, hRS7 IgG [6, 7]. This anti-Trop-2 antibody is conjugated to SN-38, the active metabolite of irinotecan, *via* a hydrolysable linker with an average drug to antibody ratio of 7.6 [6, 8]. Trop-2 is a 46 KDa transmembrane glycoprotein that is overexpressed on many solid tumor types and is correlated with an overall poor prognosis in patients, making it an attractive target for therapy [7, 9]. SG demonstrated significant clinical benefit across a range of solid tumors, including metastatic TNBC (mTNBC) [10], hormone-positive breast cancer [11], small-cell lung cancer (SCLC) [12], non-small-cell lung cancer (NSCLC) [13], and metastatic urothelial carcinomas (mUC) [14, 15]. Importantly, in mTNBC, SG has recently been granted accelerated approval by the FDA for adult patients that have failed at least two prior therapies. Further, SG has been granted Fast Track Designation from the FDA for the treatment of adult urothelial cancer patients in the neoadjuvant/adjuvant, locally advanced or metastatic setting who have previously received a programmed death receptor-1 (PD-1) or programmed death-ligand 1 (PD-L1) inhibitor, and a platinum-containing chemotherapy or who are platinum ineligible and have previously received a PD-1 or PD-L1 inhibitor.

SG affects tumor growth *via* its SN-38 payload, which inhibits topoisomerase 1 (TOP1) through stabilization of the TOP1/DNA complex [16, 17]. TOP1 is an enzyme that functions to introduce transient single-stranded DNA (ssDNA) breaks during transcription and replication in order to relieve tension in the unwinding DNA, and has long been considered a viable target for chemotherapy [18]. Once stabilized by SN-38, this TOP1/DNA complex causes dsDNA breaks upon collision with the replication fork [17]. Additionally, a cell’s response to this stabilized complex is to excise it physically from DNA, resulting in the introduction of ssDNA breaks, which if left unrepaired by PARP, will progress into dsDNA breaks [19]. These dsDNA breaks are repaired mainly by one of two methods—homologous recombination repair (HRR), which repairs the DNA with more fidelity through use of an undamaged sister chromatid as a template, or by error-prone non-homologous end-joining (NHEJ) [20].

Past preclinical studies have shown SG mediates antitumor responses in different tumor types with varying levels of Trop-2 expression [6, 8, 21]. These included a squamous cell lung carcinoma tumor line having low Trop-2 surface expression (SK-MES-1 with ~30,000 surface Trop-2 molecules per cell) [8, 21], and TNBC with moderate levels (HCC1806 with ~90,000 Trop-2 molecules per cell) [6, 21]. Further, when SG was combined with PARP inhibitors in several human TNBC cell lines, synergistic growth inhibition was noted, regardless of *BRCA1/2* status. *In vivo*, both *BRCA1/2* wild-type and mutated tumors were sensitive to the combination [22]. Notably, one *BRCA1/2* wild-type TNBC tumor that had low Trop-2 expression and was proficient in HRR [23], namely MDA-MB-231 (~30,000 surface Trop-2 molecules per cell), was not responsive to this combination. In this cell line, we found that SG exposure resulted in an upregulation of several different proteins associated with HRR, including Rad51, BRCA1-associated ring domain 1 protein (BARD1), Fanconi anemia group D2 protein (FANCD2), and excision repair cross-complementing group 1 (ERCC1). However, in SG-responsive cell lines (HCC1806 and MDA-MB-468), there was no SG-mediated upregulation of these HRR-related proteins [22]. It remains unclear from these results whether the overriding mechanism for SG sensitivity in these various tumor models is Trop-2 expression or defective HRR pathways, or their combination.

Herein, we examined the HRR response in MDA-MB-231, being unresponsive to SG, including upregulation of Rad51 and levels of dsDNA breaks mediated by SG exposure, and compared it to that of SG-sensitive tumor lines (SK-MES-1 and HCC1806) to elucidate the role that this pathway plays in protecting cells from SG-mediated dsDNA breaks. Additionally, MDA-MB-231 cells transfected to express higher levels of Trop-2 were assessed *in vivo* for SG antitumor effects in comparison to parental tumors with low Trop-2 expression. These results suggest that higher Trop-2 expression correlates with SG efficacy above negative markers associated with HRR proficiency. However, this does not rule out SG being active in tumors with low Trop-2 expression and deficiencies in HRR.

## RESULTS

### SG-mediated up-regulation of Rad51 in treatment unresponsive versus sensitive tumor cells

**Figure 1:**
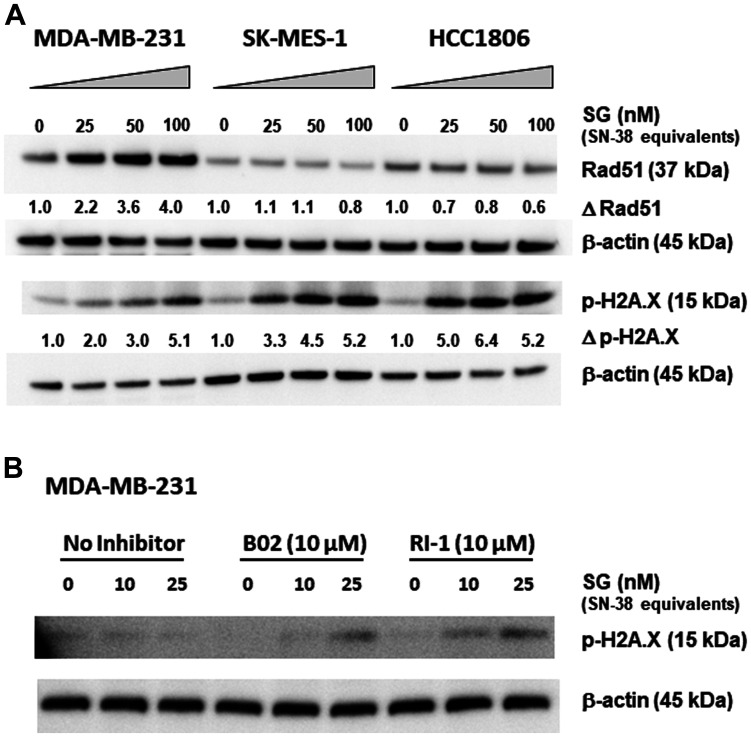
Changes in Rad51 expression and function correlate with SG-mediated resistance in MDA-MB-231. (**A**) Three different cell lines were incubated with SG for 24 h at 0, 25, 50, and 100 nM SN-38 equivalents. Assessment of changes in Rad51 expression (Δ Rad51) and dsDNA breaks (Δp-H2A.X) for each cell line was calculated as ratios relative to untreated control normalized to β-actin protein loading control. (**B**) MDA-MB-231 cells were co-incubated with SG (0, 10, and 25 nM SN-38 equivalents) plus one of two different Rad51 inhibitors (B02 and RI-1) at 10 μM for 24 h. Western blot analysis of cell lysates for both (A) and (B) was performed as described in Materials and Methods. Each experiment was performed twice.

To determine the role HRR plays in SG sensitivity, changes in Rad51 expression upon SG exposure were assessed in low Trop-2 expressing MDA-MB-231 and compared to SG-sensitive, moderate Trop-2 expressing HCC1806 and low Trop-2 expressing SK-MES-1 cell lines (Figure 1A) [24–26]. In MDA-MB-231, there was a greater than 2-fold increase in Rad51 expression after 24-h exposure to SG at the lowest concentration (25 nM SN-38-equivalents), which rose to 4-fold when incubated at the highest SG concentration (100 nM). By contrast, both SK-MES-1 and HCC1806 cells did not demonstrate any increases in Rad51 expression upon SG exposure, and in fact exhibited down-regulation at 100 nM in SK-MES-1 and at all three concentrations in HCC1806. Resulting dsDNA breaks, as evidenced by increased levels of phosphorylated histone H2A.X (p-H2A.X) [27], also were determined in these same three cell lines. At 25 nM, there was an approximate 70% increase in dsDNA breaks in SK-MES-1 and 155% increase in HCC1806 compared to MDA-MB-231. This increase in p-H2A.X observed in both SK-MES-1 and HCC1806 cells relative to MDA-MB-231 continued at the 50 nM concentration of SG (50% and 114%, respectively). Only at the highest concentration of SG (100 nM) was there an equivalent amount of DNA damage among the three cell lines. Taken together, these data suggest that while a tumor cell less sensitive to SG may be proficient in activating HRR-mediated pathways, as evidenced by increased Rad51 expression and lower levels of dsDNA breaks, there is a threshold of SN-38-mediated damage above which cells are unable to maintain adequate DNA repair, resulting in increased dsDNA breaks and likely pushing cells towards apoptosis.

To confirm the role of HRR in imparting SG resistance to MDA-MB-231, cells were incubated with two different Rad51-inhibitors, B02 and RI-1 [28, 29]. In the absence of inhibitors, the two concentrations of SG tested (10 and 25 nM SN-38-equivalents) resulted in no detectable dsDNA breaks (i.e., p-H2A.X levels remained unchanged relative to untreated cells; Figure 1B). Likewise, neither B02 nor RI-1, when used alone, had any effect on the cells, as evidenced by no changes to the amount of p-H2A.X levels relative to untreated control cells. However, when combined with SG at 25 nM SN-38-equivalents, cells incubated with either B02 or RI-1 demonstrated noticeably higher levels of dsDNA breaks compared to baseline controls. Of the two inhibitors, RI-1 appears to be more potent, since SG concentrations as low as 10 nM SN-38-equivalents produced detectable levels of p-H2A.X. These data demonstrate that by inhibiting the normal functioning of Rad51, the SG low-responsive MDA-MB-231 became more sensitive to the DNA-damaging effects of SG, thus indicating an important role that HRR-pathways play in mitigating the activity of SG.

### *In vitro* and *in vivo* characterization of MDA-MB-231 Trop-2-transfection clones C13 and C39

**Figure 2:**
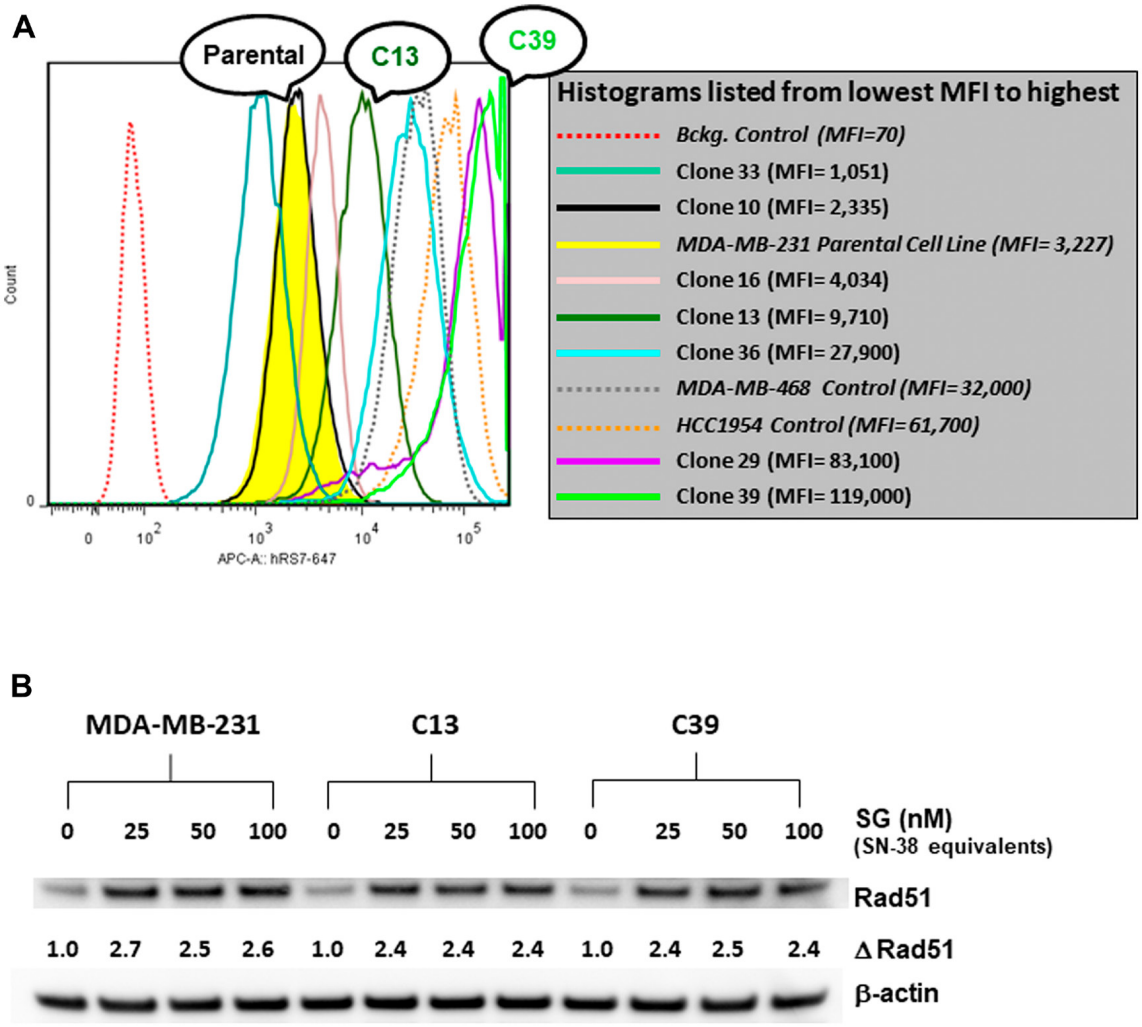
FACS analysis of various MDA-MB-231 Trop-2-transfectants and assessment of SG-mediated changes in Rad51 expression. (**A**) MDA-MB-231 was transfected with human Trop-2 as described in Materials and Methods. After G418 selection, seven clones were isolated and analyzed for surface expression of Trop-2 *via* FACS. Two cell lines with high Trop-2 expression (MDA-MB-468 and HCC1954) were used as positive controls. Parental MDA-MB-231 histogram shaded in yellow. MFI = mean fluorescence intensity. (**B**) Parental MDA-MB-231 and clones C13 and C39 were incubated with SG at the indicated concentrations for 24 h. Cell lysates were analyzed by western blot as described in Materials and Methods. Assessment of changes in Rad51 expression (Δ Rad51) for each cell line was calculated as ratios relative to untreated control normalized to β-actin protein loading control.

The possible role of Trop-2 as a positive biomarker for SG sensitivity was assessed in MDA-MB-231 tumor cells with different amounts of Trop-2 surface expression. First the cDNA of human Trop-2 (GenBank: X77754.1) was transfected into MDA-MB-231 cells [30]. After G418 selection, FACS analysis identified 5 clones that exhibited Trop-2 expression levels above that observed for MDA-MB-231 parental cells (Figure 2A). Of the 5, two clones, C13 and C39, were selected for further experimentation. Growth of these two clones, as well as the parental MDA-MB-231 cell line in G418-free media for over 6 months, demonstrated that this higher Trop-2 expression was stable (Table 1). Clone C13 expressed >3.5-fold higher Trop-2 than parental MDA-MB-231, while clone C39 expressed >24-fold higher levels. Further, baseline levels of Rad51 in both clones were similar to parental levels (Figure 2B). In terms of HRR response to SG exposure, the up-regulation of Rad51 was comparable for both clones and the parental MDA-MB-231 (Figure 2B). This suggests that increased expression of Trop-2 did not have an impact on SG-induced HRR in either of these high Trop-2-expressing clones.

**Table 1:** 6-month stability of Trop-2 expression in MDA-MB-231 parental cells and Clones (C13 and C39)

Number of Surface Trop-2 Molecules per Cell (days post-cloning)				
**Cell Line**	**(0 days)**	**(105 days)**	**(168 days)**	**Mean ± s.d.**
**Parental**	36,592	21,257	37,760	31,870 ± 9,209
**Clone C13**	93,864	113,151	155,225	120,747 ± 31,378
**Clone C39**	563,418	991,016	812,291	788,908 ± 214,756

**Figure 3:**
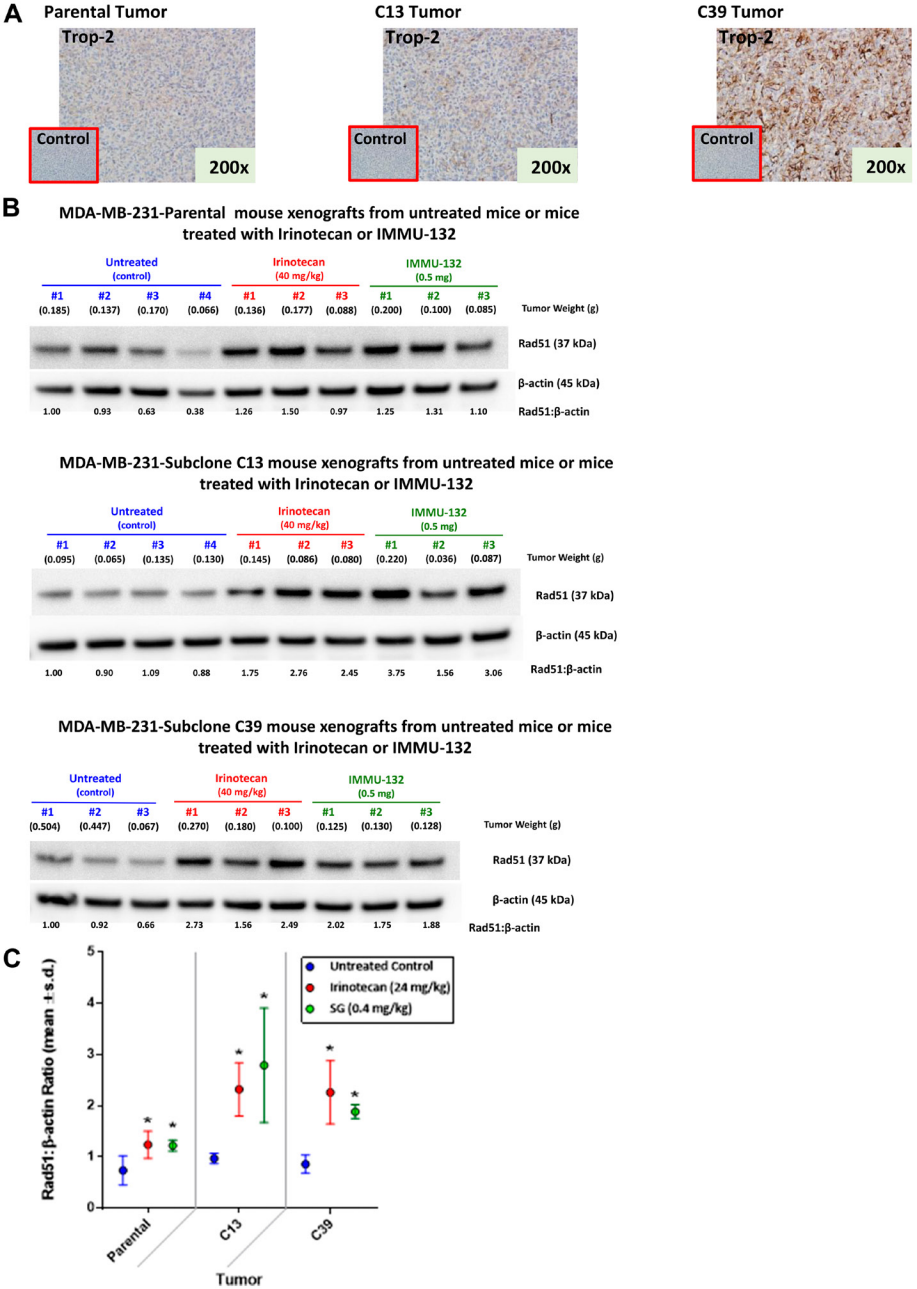
Expression of Trop-2 and changes in Rad51 expression mediated by irinotecan and SG in tumor xenografts of parental MDA-MB-231 and clones 13 and 39. MDA-MB-231 tumors as well as those grown from C13 and C39 cells were established in NCr *nu*/*nu* mice as described in Materials and Methods. (**A**) Tumors were removed from mice and formalin-fixed prior to IHC staining with a goat anti-human Trop-2 polyclonal antibody as described in Materials and Methods. Negative control staining was with normal goat antibody. (**B**) Mice bearing parental MDA-MB-231, C13, or C39 tumors were injected i.v. with either irinotecan or SG (doses shown in SN-38 equivalents). Mice that received no therapy served as untreated control. After 24 h, mice were euthanized and tumors removed and flash-frozen. Frozen tumors were analyzed for Rad51 *via* Western blot as described in Materials and Methods. Relative ratio of Rad51 to β-actin loading control was based on untreated Animal 1 for each tumor type. The ratio for this mouse was set at 1.0 with all other ratios relative to this animal for each tumor type. (**C**) Mean Rad51:β-actin ratios for all the mice within a treatment group for each tumor-type (^*^ *P* ≤ 0.05; one-tailed *t*-Test).

IHC of FFPE parental MDA-MB-231, C13, and C39 tumor xenografts confirmed greater Trop-2 expression in C13 relative to parental MDA-MB-231 with an even greater staining in C39, demonstrating that the higher Trop-2 levels in the clones is maintained *in vivo* (Figure 3A). Similar to *in vitro* observations, mice bearing parental MDA-MB-231, C13, or C39 tumors treated with SG demonstrated up-regulation of Rad51 within 24 h post-injection (Figure 3B). Likewise, tumors had a similar response in mice treated with irinotecan. A comparison between tumors taken from either SG- or irinotecan-treated mice with those from untreated animals showed that this increase in Rad51 was significant (Figure 3C; *P* ≤ 0.05). However, there were no significant differences in Rad51 expression between mice treated with SG and those treated with irinotecan. These data indicate that HRR activation in response to SG and irinotecan in the mice bearing tumors derived from either of the two high Trop-2 clones were similar to those in mice bearing the parental MDA-MB-231 tumors.

### Changes in SG efficacy in C13- and C39-derived tumors versus parental MDA-MB-231

**Figure 4:**
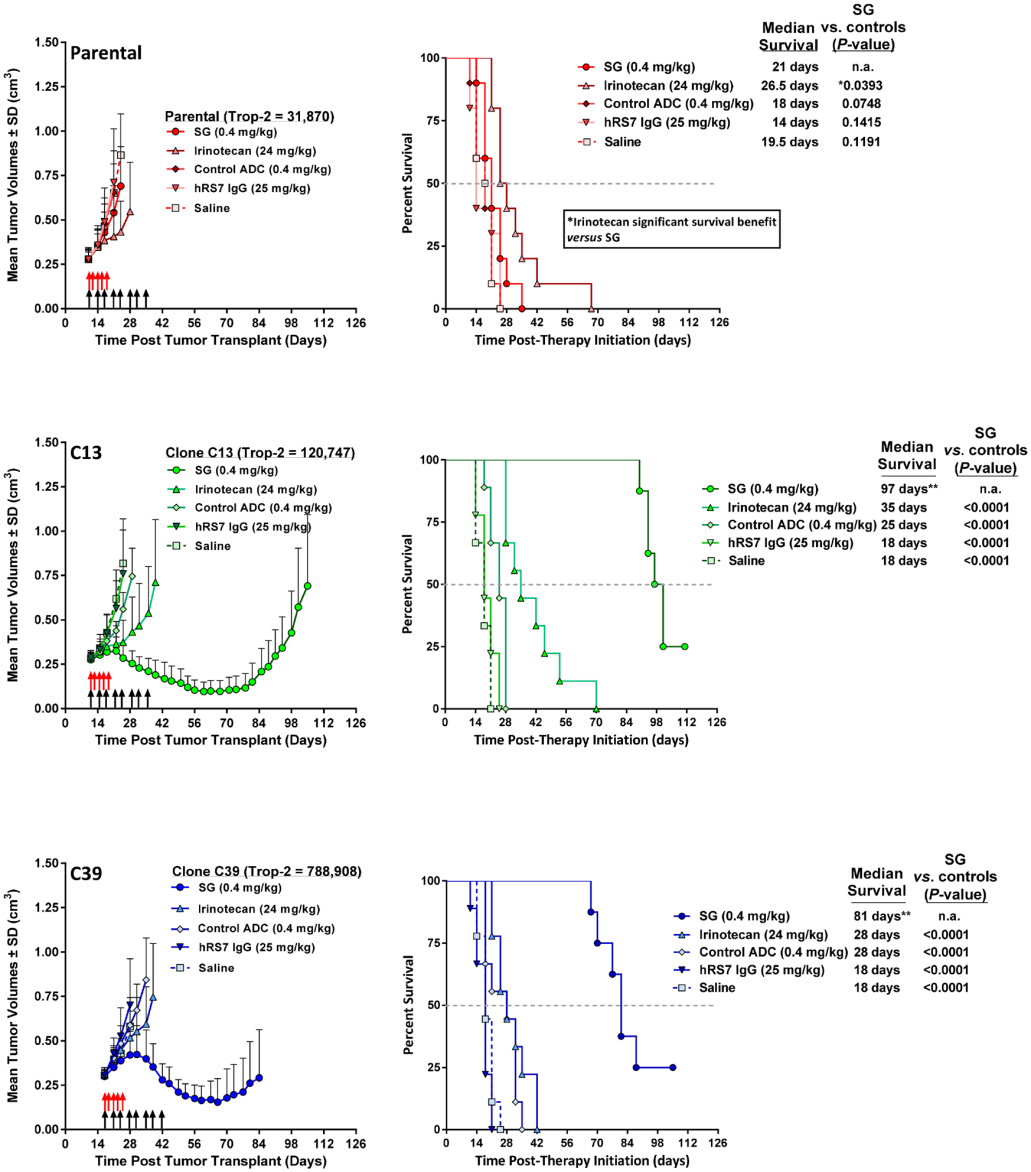
Increased Trop-2 expression in MDA-MB-231 tumors overcomes resistance to SG but not irinotecan. NCr athymic *nu*/*nu* mice were injected s.c. with either MDA-MB-231 parental cells (parental), MDA-MB-231 clone 13 (C13) cells, or MDA-MB-231 clone 39 (C39) cells as described in Materials and Methods. Once tumors reached ~0.3 cm^3^ in size, mice were randomized into the various treatment groups. SG, control ADC, and parental hRS7 IgG antibody, were administered i.p., twice weekly for 4 weeks (black arrows). Irinotecan was administered i.v. at its MTD (q2dx5; red arrows). For all animal studies, the doses of SN-38 immunoconjugates and irinotecan are shown in SN-38 equivalents. The dose of hRS7 is shown at its protein dose equivalent to SG protein dose. Graphs to the left show mean tumor growth curves for each treatment group while those on the left indicate survival curves for these same groups of animals. ^**^One mouse in SG group deemed an outlier *via* Grubbs’ test and removed from final analysis. Grey dotted line in survival curves indicates 50% survival line.

Mice bearing parental MDA-MB-231-, C13-, or C39-derived tumor xenografts were treated with SG (25 mg/kg twice weekly for 4 weeks; 0.4 mg/kg SN-38 equivalents) or the maximum tolerated dose (MTD) of irinotecan (40 mg/kg q2dx5; 24 mg/kg SN-38 equivalents). Growth rates for saline control tumors for all three tumor types were not significantly different from each other (MST = 19.5d, 18d, and 18d for parental, C13, and C39 tumors, respectively; Figure 4). In all three tumor types, irinotecan at its MTD provided a significant survival benefit relative to saline control animals, with MSTs ranging from 26.5 days in mice with parental tumors to 35 and 28 days in mice with C13 and C39 tumors, respectively (*P* < 0.0009). There were no significant differences in survival between mice bearing parental, C13, or C39 tumors when treated with irinotecan, indicating that we have not increased the sensitivity of either C13 or C39 tumors to the effect of irinotecan by increasing Trop-2 expression levels. As expected, SG was ineffective in mice bearing parental MDA-MB-231 tumors (MST = 21d). Further, in those animals bearing parental tumors, irinotecan provided a significant survival benefit in comparison to animals treated with SG (*P* = 0.0393). Similar to SG, a non-specific control ADC did not produce significant antitumor effects in animals bearing the parental tumor. However, in mice bearing C13 and C39 tumors, the non-specific ADC did slow tumor growth relative to saline controls (MST = 25d and 28d, respectively; *P* < 0.0158 *vs*. saline), but was significantly less effective than irinotecan in C13 (*P* < 0.0009) and no different than irinotecan in C39 tumor-bearing animals. Most importantly, unlike the parental tumors, where the average tumor size showed no evidence of response to SG treatment (i.e., tumor size reduction), the average tumor volume in animals bearing the C13 and C39 clones decreased greater than 52% from their initial tumor sizes upon SG treatment. These tumor regressions continued for more than three weeks after the final SG injection was administered to the animals. This translated into a greater than 2.7-fold increase in MST in each model compared with all other treatments, including irinotecan and control ADC therapy (MST = 97d and 81d for SG treated C13 and C39 tumors, respectively; *P* < 0.0001 *vs*. all control groups). It is important to note that mice administered irinotecan received 37.5-fold more SN-38 than those treated with SG (2.4 mg *vs.* 0.064 mg total SN-38 equivalents, respectively), suggesting that the improved efficacy observed in the C13 and C39 tumor-bearing mice was likely due to increased SN-38 targeting and uptake mediated by SG due to higher Trop-2 expression and not to any gained sensitivity to SN-38 itself. Finally, despite C39 tumors having approximately 6.5-fold more Trop-2 expression than C13 tumors, there was no significant difference in antitumor effects between the two when treated with SG, suggesting that once a certain threshold of SN-38 delivery to the tumor is reached, maximum DNA damage is inflicted on the cells, and addition of more SN-38 above this will not enhance the effects.

### DISCUSSION

As ADCs become approved for the treatment of solid tumors [31], the utilization of biomarkers to predict therapeutic outcome will require additional interrogation, including the antigen target of the antibody, as well as the sensitivity to the drug-payload. For ADCs, the primary biomarker will always be the tumor antigen bound by the antibody moiety. However, while it is important for the tumor to express the targeted antigen, the relative expression levels and accessibility of that antigen may also need to be considered in terms of response. Clinically, SG, with its Trop-2-binding monoclonal antibody coupled to an SN-38 payload, has demonstrated efficacy in a range of solid tumors, including mTNBC, for which it recently gained accelerated approval in adult patients who have received at least two prior therapies for metastatic disease [10–15]. A phase III trial in hormone receptor-positive breast cancer, TROPiCS-02 (NCT03901339), is also underway. Promising results have also been observed in the mUC cohort of the IMMU-132-01 basket trial (NCT01631552), and clinical activity has been confirmed recently in a phase II trial in this patient population leading to the FDA granting Fast Track Designation (TROPHY U-01 study; NCT03547973) [14, 15]. Additionally, a phase II study (TROPiCS-03; NCT03964727) is currently ongoing in patients with metastatic solid tumors selected based on elevated Trop-2 expression by a validated IHC assay.

Here we examined the potential role of biomarkers in predicting the efficacy of SG. Trop-2 expression levels as a positive, primary biomarker and HRR proficiency as a secondary, negative biomarker were assessed *in vitro* and *in vivo*. In two different tumor lines with similar, low Trop-2 expression levels, the less SG-sensitive cell line (MDA-MB-231) readily up-regulated Rad51 in response to SG exposure, while the SG-sensitive cell line (SK-MES-1) was defective in HRR, as demonstrated by the lack of Rad51 up-regulation. Moreover, when Trop-2 expression levels were increased in MDA-MB-231, this previously less-responsive tumor was rendered more sensitive to SG therapy *in vivo,* as evidenced by tumor regressions and significantly improved survival benefit, which were greater than those achieved with irinotecan treatment.

This approach of testing for a positive therapeutic correlation between expression levels of a given biomarker and an ADC has been examined by others [32–34]. In one phase III study examining biomarkers in patients with either locally advanced or metastatic breast cancer (mBC) previously treated with ≥ 2 anti-HER2-directed therapies (trastuzumab and lapatinib), treatment with the anti-HER2 ADC, trastuzumab emtansine (T-DM1), produced a numerically greater progression-free survival (PFS) benefit *vs* therapy of physician’s choice (TPC) in those patients whose tumors had greater than median levels of HER2 mRNA [32]. Further analysis of the T-DM1 treatment subgroup of patients likewise showed a numerically longer PFS in those patients with higher than median levels of HER2 mRNA expression compared to T-DM1 treated patients with less than or equal to median HER2 expression. Whereas it appears that high HER2 expression levels correlated with improved PFS and overall survival in T-DM1-treated patients, the underlying mechanisms of action of T-DM1 fundamentally differ from those ascribed to SG. Given the stable thioether linker used by T-DM1, only targeted cells, and subsequent uptake by these cells, is required to release the drug [35]. Under such constrained drug-delivery conditions, it follows that the amount of drug delivered by T-DM1 is strictly related to the amount of HER2 expressed by the tumor cells. By contrast, SG will release its SN-38 payload both intra- and extra-cellularly in the tumor microenvironment due to its hydrolysable CL2A linker [6, 21]. Collectively, the release of SN-38 from both tumor cell-bound and internalized SG, as well as within the tumor microenvironment of non-internalized or unbound SG, may deliver more than enough SN-38 to allow for significant tumor-cell death in both high and low Trop-2 expressing tumors. In agreement with this, past preclinical studies have shown SG to mediate specific antitumor responses in different tumor types with varying levels of Trop-2 expression [6, 8, 21]. However, in MDA-MB-231 TNBC, a tumor line with low levels of Trop-2 (~30,000 molecules per cell), low responses to SG were noted in tumor-bearing mice, whereas treatment with irinotecan resulted in a modest, but significant, inhibition of tumor growth [6, 21]. Further, in HCC1806 TNBC tumors, with 3-fold higher Trop-2 levels (~90,000/cell), SG mediated significant tumor regressions [21, 22]. This suggests that higher Trop-2 levels would predict SG sensitivity. However, in SK-MES-1 human squamous cell lung carcinoma cells with similar Trop-2 expression levels as MDA-MB-231 (~30,000), both SG and irinotecan provided significant antitumor effects [8, 21]. These data indicate that other biomarkers, in addition to Trop-2, may play a role in SG-mediated antitumor responses.

In the SG-resistant, *BRCA1/2*-wildtype MDA-MB-231 TNBC tumor line, several HRR proteins were found to be upregulated upon SG exposure, among them ERCC1 and Rad51 [22]. ERCC1 forms a heterodimer with the protein coded for in the *ERCC4* gene (XPF) to produce the ERCC1-XPF nuclease, which plays a role in several DNA repair pathways, including nucleotide excision repair as well as in dsDNA break repair [36]. Together with PARP, ERCC1-XPF participates in repairing DNA damage mediated by TOP1 inhibitors through the excision of the stabilized TOP1/DNA complex and subsequent repair of the resulting ssDNA break [19]. Of note, Rad51 expression has been recognized as a predictable biomarker for HRR proficiency [25]. In addition to its role in HRR, Rad51 also plays a role in replication fork protection that is independent of BRCA2 and whose activity would also help protect the cell from SN-38-mediated replication fork collapse [37, 38]. Consistent with this, we too found that lack of Rad51 upregulation by both SK-MES-1 and HCC1806 SG-sensitive tumor lines correlates with known SG *in vivo* sensitivity [6, 8]. Further, as the SN-38 concentrations increased, the amount of Rad51 was reduced in both cell lines. This drop in Rad51 was likely due to the increased DNA damage in these sensitive cells, resulting in a negative impact on protein synthesis [23]. However, for the SG-poorly-responsive MDA-MB-231 tumor line, Rad51 levels rose greater than 2-fold within 24 h of SG exposure. These results also correlated with the degree of dsDNA breaks mediated by SG in all three of these tumor lines, with low levels of SG mediating greater amounts of dsDNA breaks in those tumor lines that did not upregulate Rad51 compared to MDA-MB-231. Only when the MDA-MB-231 cells were treated with Rad51-inhibitors was an increase in dsDNA breaks noted in the SG treated cells compared to cells not treated with these inhibitors.

It should be noted that MDA-MB-231 is a well-characterized tumor line with known proficiency in HRR pathways [23, 29, 39–42]. When assessing clinical tumor specimens, however, expression levels of various proteins associated with activation of these HRR pathways may not be sufficient to truly detect the functionality of HRR to repair-damaged DNA. Current efforts for detecting HRR defects as well as other potential biomarkers center on proteomics, RNA-sequencing, and qualitative next-generation sequencing (e.g., FoundationOne^®^CDx) [1–5]. Likewise, functional assays are being developed to measure the ability of tumor tissue to actively produce RAD51/DNA foci as an indication of HRR efficiency [26, 27, 43]. Results from such assays would provide a better indication as to whether a given patient’s tumor would respond to a DNA-damaging chemotherapeutic *versus* some other targeted therapy.

In addition to HRR, a patient’s tumor may express other biomarkers associated with SN-38 responses. One such predictor of SN-38 sensitivity is Schlafen-11 (SLFN11), whose enhanced expression is linked to increased sensitivity to TOP1 inhibitors [44]. SLFN11 is recruited to stressed replication forks, where it blocks fork progression independent of ataxia telangiectasia and Rad3-related protein (ATR) [45]. MDA-MB-231 has low SLFN11 levels consistent with resistance to TOP1 inhibitors [44]. Nevertheless, as with HRR, SLFN11-mediated resistance was overcome in high Trop-2 MDA-MB-231 tumors, showing the prominence of Trop-2 expression over this negative biomarker (i.e., low SLFN-11 expression). In terms of biomarkers associated with acquired resistance to irinotecan, SG may be unable to overcome such a barrier, but this needs further study.

Acquired resistance can be multifaceted because a patient’s tumor may have developed multidrug resistance through ABCG2 or through mutation of TOP1 [46, 47]. Loss of SN-38 activity through the tumor cell’s lack of dependence on TOP1 would make further treatment with SG futile [46, 47]. Further, we had previously demonstrated that SG is unable to overcome SN-38 resistance mediated through the ABCG2 multidrug resistance pump [48]. Even in a high Trop-2 expressing tumor (~250,000 Trop-2 molecules per cell), SG was unable to overcome ABCG2-mediated resistance *in vivo* [48]. For such reasons, patients previously treated with irinotecan are typically excluded from SG therapy. However, SG may still have activity in those patients who never responded to this chemotherapeutic if it was due to efficient DNA damage repair, low SLFN11 levels, or poor conversion of irinotecan to SN-38. If such patients present with high Trop-2-expressing tumors, they may still respond to SG therapy and could be considered for treatment. Indeed, in a phase I/II SG basket trial (NCT01631552), of 9 patients that had previously failed therapies consisting of TOP1 inhibitors, 2 demonstrated significant tumor regressions of target lesions after SG treatment, 5 had stable disease, while the remaining 2 patients progressed during treatment [49]. While it is not known why these patients ultimately failed to respond to their initial TOP1 inhibitors, delivery of SN-38 to these same patients by SG was able to overcome some of these resistance mechanisms.

While these data explain the difference in SG sensitivity between cells with active DNA damage repair (DDR) pathways and those with impaired pathways in low Trop-2-expressing tumor cells, they do not separate these two potential biomarkers in terms of superiority. We demonstrate that in high Trop-2 expressing tumors, SG causes a significant amount of DNA damage resulting in tumor regression, despite functioning HRR pathways. Conversely, at low Trop-2 expression levels, the cellular response to the DNA damage mediated by SG takes precedence over Trop-2 expression. Importantly, there may be a threshold of SN-38-mediated damage above which even HRR proficient tumor cells are unable to repair the damage. This was noted *in vitro* in MDA-MB-231 cells in which there was a threshold at which the amount of DNA damage in this poorly responsive tumor line equaled that of the two SG-sensitive lines when exposed to greater SN-38 levels. We found that in mice bearing MDA-MB-231 tumors with surface Trop-2 expression greater than 4-fold higher than parental tumor cells, SG therapy resulted in tumor regression with significant survival benefit observed despite having intact and functioning DDR pathways. Further, this antitumor effect was significantly greater than that achieved with irinotecan. We have previously demonstrated that SG delivers higher amounts of SN-38 to tumor xenografts compared to irinotecan even when mice were administered 28-fold more SN-38 equivalents of irinotecan compared to SG [50]. Since mice can efficiently convert irinotecan to SN-38 due to the presence of the requisite carboxylesterase in their serum [51], mice treated with irinotecan in this current study were administered greater than 36-fold more SN-38 equivalents than those treated with SG. However, despite this advantage, and consistent with our previous findings, it is likely that this significantly improved antitumor effect observed in the SG-treated mice with high Trop-2 expression was due to a greater concentration of SN-38 maintained in the tumor compared to that which was achieved with irinotecan. This difference between SG and irinotecan therapy should prove to be more relevant clinically because humans are not as efficient as mice in converting irinotecan to SN-38; indeed, plasma area-under-the-concentration *vs* time-curve values for SN-38 are 2% to 8% of those for irinotecan in patients [52]. These data suggest that there is a minimum threshold of SN-38-mediated damage above which HRR fails to adequately compensate, resulting in catastrophic damage to the DNA and ultimately tumor cell death. Furthermore, unlike irinotecan, SG can surpass this threshold due to its efficient delivery of SN-38 to these high Trop-2-expressing tumors.

To conclude, these data strongly support the hypothesis that as a biomarker, high surface Trop-2 expression on a patient’s tumor may be predictive of a positive clinical outcome for SG therapy. Further, there are secondary biomarkers that may need to be considered for those patients with low/moderate Trop-2 expression or those with high Trop-2 expression that failed previous irinotecan therapy for reasons other than acquired resistance. Moreover, while high expression of Trop-2 was found to be a primary biomarker for SG efficacy, it should not be a limiting factor, because other secondary biomarkers coupled with Trop-2 expression may likewise be predictive of clinical benefit. For these reasons, future clinical trials will need to comprehensively examine potential biomarkers, in addition to Trop-2 expression, to generate a profile that will better identify those patients likely to benefit from SG therapy.

### MATERIALS AND METHODS

### Cell lines, antibody-drug conjugates, and antibodies

Human TNBC (MDA-MB-231, HCC1806, and MDA-MB-468), HER2+ breast cancer (HCC1954), and squamous cell lung carcinoma (SK-MES-1) cell lines were purchased from the American Type Culture Collection (ATCC; Manassas, VA, USA). Each was maintained according to the recommendations of ATCC, in culture less than 6 months, and routinely tested for mycoplasma using MycoAlert^®^ Mycoplasma Detection Kit (Lonza; Rockland, ME, USA). Any cell line with an unknown passage number was authenticated by short tandem repeat (STR) assay by the ATCC. SG, control ADC (h679-CL2A-SN-38; anti-histamine-succinyl-glycine), and hRS7 IgG were prepared by Immunomedics, Inc. (Morris Plains, NJ, USA). For *in vitro* assays, SG is expressed in terms of SN-38 equivalents. For example, based on a mean SN-38/IgG substitution ratio of seven, a concentration of 14.3 nM SG would be equivalent to 100 nM SN-38. For animal studies a 500 μg dose of SG to a 20-g mouse (25 mg/kg) would contain 0.46 mg/kg of SN-38. Irinotecan doses are likewise shown as SN-38 equivalents (i.e., 40 mg irinotecan/kg is equivalent to 24 mg/kg of SN-38).

### Assessment of Trop-2 expression in transfected MDA-MB-231 and tumor xenografts

MDA-MB-231 was transfected with human Trop-2 and subcloned as described previously [30]. Quantification of surface Trop-2 expression as determined by FACS analysis, as well as expression on tumor xenografts by immunohistochemistry (IHC) of formalin-fixed, paraffin-embedded (FFPE) tissues, was done as described previously [21]. Selected clones expressing moderate (clone C13) and high (clone C39) Trop-2 levels were cultured for at least 6 months in selection-free media before use to ensure stable expression of Trop-2. MDA-MB-468 and HCC1954 tumor lines, with known Trop-2 expression levels (~300,000 and ~650,000 molecules per cell, respectively) were used as controls [21].

### Western blot assessment of Rad51 expression and dsDNA breaks *in vitro*

Cells (MDA-MB-231, SK-MES-1, or HCC1806) were plated overnight in 6-well plates. The following day, SG (0, 25, 50, or 100 nM SN-38-equivalents) was added to appropriate wells for 24 h. For Rad51 expression, cells were harvested, lysed and protein concentrations determined using BCA Protein Assay Kit (Thermo Fisher; Grand Island, NY, USA). A total of 25 μg protein was resolved in a 4–12% Bis-Tris NuPAGE gels and transferred to a polyvinylidene difluoride (PVDF) membrane. For phosphorylated-H2A.X (pH2A.X), 50 μg total protein was loaded followed by transfer to a nitrocellulose membrane. Blots were blocked with 5% nonfat milk in 1× TBS-T for 1 h at room temperature. Membranes were probed overnight at 4°C with primary antibody, followed by 1 h incubation at room temperature with secondary antibody. Rabbit anti-human primary antibodies were purchased from Cell Signaling Technology (Danvers, MA, USA), and included anti-Rad51 (Cat. No. 8875), anti-phospho-H2A.X (Cat. No. 2577), and anti-β-actin (Cat. No. 4967). Secondary antibody was horseradish peroxidase-conjugated antibody to rabbit (Jackson Immunoresearch; West Grove, PA, USA; Cat. No. 111-035-046). SignalFire™ ECL Reagent (Cell Signaling Technology) was used for detection.

### *In vivo* therapy studies

All animal studies were approved by Montclair State University Institutional Animal Care and Use Committee. Tumor xenografts of parental MDA-MB-231, as well as for clones C13 and C39, were established by harvesting cells from tissue culture and mixing 1:1 with matrigel and injecting cells into the flank of NCr athymic nude mice (Taconic; Germantown, NY, USA), such that each mouse received ~1 × 10^7^ cells. Tumor volume was determined by measurements in two dimensions using calipers, with volumes defined as: *L* × *w*^2^/2, where *L* is the longest dimension of the tumor and *w* the shortest. Mice were randomized into treatment groups and therapy begun when tumor volumes were approximately 0.3 cm^3^. Mice were euthanized once tumors grew to greater than 1.0 cm^3^ in size.

All treatment regimens, dosages, and number of animals in each experiment involving the three different tumor types (i.e., parental, C13, and C39) are described in the Results and figure legends. Irinotecan hydrochloride (AREVA Pharmaceuticals, Inc., Georgetown, IN, USA) was diluted in sterile saline (0.9% NaCl, Injection, USP; Hospira, Inc., Lake Forest, IL, USA) before injection. Likewise, lyophilized SG and control ADC were reconstituted and diluted as required in sterile saline.

### Western blot assessment of Rad51 expression *in vivo*

MDA-MB-231, C39, and C13 tumors were established in mice as described above. Once tumors reached approximately 0.3 cm^3^ in size, animals were randomized into treatment groups (*N* = 3–4). Tumor-bearing mice were treated with either irinotecan (40 mg/kg; 24 mg/kg SN-38 equivalents) or SG (25 mg/kg; 0.4 mg/kg SN-38 equivalents). One group of animals for each tumor type was left untreated as controls. To be consistent with the *in vitro* experiments, after 24 h, the mice were euthanized, and the tumors removed and flash-frozen. Tumors were homogenized on ice in RIPA buffer (Pierce Thermo Scientific, cat no. 89900) plus Protease/Phosphatase Inhibitor Cocktail (100×, Cell Signaling Technology, cat. no. 5872S). Homogenates were placed on a sample rotator for 2 h at 4°C, followed by centrifugation (12,000× g) for 20 min at 4°C. Protein concentrations of the supernatant and subsequent western blot analyses were performed as described above.

### Statistical analysis of *in vivo* data

A Grubbs’ test was performed on the data from treatment and control groups with *P* ≤ 0.05 for any mouse deemed an outlier. Such mice were removed from further statistical analysis and are noted in the Results. Survival studies were analyzed using Kaplan–Meier plots (log-rank analysis), using the Prism GraphPad Software package (v7.02; Advanced Graphics Software, Inc.; Encinitas, CA, USA). Significance was set at *P* ≤ 0.05.
